# A model integrating tonic and antigen-triggered BCR signals to predict the survival of primary B cells

**DOI:** 10.1038/s41598-017-13993-x

**Published:** 2017-11-02

**Authors:** Shoya Yasuda, Yang Zhou, Yanqing Wang, Masayuki Yamamura, Ji-Yang Wang

**Affiliations:** 1 Department of Computational Intelligence and Systems Science, Tokyo Institute of Technology, Yokohama 226-8502, Japan; 2 Department of Integrative Medicine and Neurobiology, State Key Laboratory of Medical Neurobiology, Institutes of Brain Science, Collaborative Innovation Center for Brain Science, Fudan University, Shanghai 200032, China; 3 School of Computing, Tokyo Institute of Technology, Yokohama 226-8502, Japan; 4 Department of Immunology, School of Basic Medical Sciences, Fudan University, Shanghai 200032, China

## Abstract

The BCR constitutively transmits a “tonic” survival signal in the absence of exogenous antigen-binding. However, the strength of tonic BCR signal and its relationship with antigen-triggered survival signal are poorly understood. We found that primary B cells expressing high levels of BCR had elevated BCR tonic signal and increased survival compared with those expressing low levels of BCR. In addition, we found that crosslinking BCR with low doses of F(ab′)_2_ α-IgM antibodies did not enhance, but rather decreased, B cell survival and that only when most of the BCR were occupied by F(ab′)_2_ α-IgM antibodies was B cell survival enhanced. Based on these experimental results, we present a mathematical model integrating tonic and antigen-triggered BCR signals. Our model indicates that the signal generated from crosslinked BCR is 4.3 times as strong as the tonic signal generated from free BCR and that the threshold of B cell activation corresponds to the signal generated by crosslinking 61% of the surface BCR. This model also allows the prediction of the survival probability of a B cell based on its initial BCR level and the strength and duration of antigen stimulation, and fits with the mechanism of B cell tolerance.

## Introduction

The B cell receptor (BCR) is a heterotrimeric complex consisting of antigen (Ag) binding immunoglobulins and the signal-transducing Igα/Igβ heterodimers. In mature B cells, Ag binding to the BCR initiates a cascade of signaling events that eventually lead to the activation of transcription factors such as NF-κB, NFAT and AP-1, which regulates the expression of genes involved in B cell survival, activation and differentiation^1–3^. Dysregulated BCR signaling results in altered survival and activation of B cells and B cell-mediated immune responses, leading to primary immunodeficiencies^4,5^, autoimmune diseases^6–9^ and even B cell malignancies^10,11^. It is therefore important to understand the mechanisms by which the exogenous Ag stimulation is converted to the survival and activation signals. Studies thus far have revealed many tyrosine kinases and adaptor molecules that participate in BCR signal transduction triggered by BCR stimulation^12^. Both negative^13^ and positive^14^ feedback mechanisms that regulate BCR signaling have been identified. Whereas the negative feedback system functions to prevent excessive signals, the positive feedback mechanism can result in a steep dose response to Ag stimulation and can thus function as an on/off switch of signal transduction. An intriguing feature of BCR signaling is that there is an activation threshold^14–16^. In other words, while B cells do not respond to low doses of Ag stimulation, a robust response can be induced when the Ag dose reaches a certain level. The existence of such a threshold can be explained in part by a positive feedback mechanism in the regulation of NF-κB activation^14^. The presence of a threshold in Ag-triggered BCR signaling functions to prevent B cell activation by self Ag, which binds to autologous B cells only weakly, and is an important mechanism for maintaining peripheral B cell tolerance.

Although BCR signal transduction has been extensively studied thus far, most studies have focused on exogenous Ag-triggered BCR signaling events. It is now clear that, even in the absence of Ag binding, BCR constitutively transmits a tonic survival signal. The requirement of tonic BCR signal for B cell survival has been demonstrated by the finding that *in vivo* ablation of BCR expression in mice causes rapid death of B cells^17^. The tonic BCR survival signal is transmitted through Igα and Igβ heterodimers^18^ and the B cell death due to the lack of tonic BCR signal can be rescued by PI3 kinase signaling^19^. These results provide compelling evidence that BCR transmits a tonic signal in the absence of Ag stimulation though Igα and Igβ heterodimers and activates the downstream PI3 kinase to maintain B cell survival. Further studies have revealed that tonic BCR signal is also important for the survival of malignant B cells^20^ even though these B cells have oncogenic mutations that lead to their uncontrolled proliferation. Despite the biological significance of tonic BCR signal, it is difficult to analyze its signaling events in detail using conventional biochemical or immunological approaches. The strength of the intrinsic tonic BCR signal and its relationship with the extrinsic Ag-triggered survival signal remain largely unknown.

We decided to address the regulation of tonic signal by analyzing the kinetics of B cell survival during *in vitro* culture in the absence of exogenous Ag stimulation. In addition, to investigate the possible interactions between tonic and Ag-triggered BCR signal, we have analyzed the kinetics of B cell survival in response to a wide range of doses of F(ab′)_2_ α-IgM antibodies (Abs), which mimic Ag stimulation. We found that B cell survival in the absence of Ag stimulation positively correlated with BCR levels. In addition, we found that F(ab′)_2_ α-IgM Abs enhanced B cell survival only when most of the cell surface BCR were crosslinked by these Abs. Based on these and additional experimental results, we provide a mathematical model integrating the tonic BCR signal and Ag-triggered survival signal. This model reveals the relative strength of the tonic signal generated from free BCR and the signal generated from crosslinked BCR. This model also allows us to predict the survival probability of a primary B cell cultured under various conditions and provides new insights into the mechanisms of B cell tolerance.

## Materials and Methods

### Mice

C57BL/6 mice were purchased from Shanghai Slac Laboratory Animal Corporation and maintained in specific pathogen free conditions. All animal experiments were approved by Fudan University Animal Committee. In addition, all methods were performed in accordance with the guidelines and regulations of Fudan University.

### B cell culture and survival assay

Primary B cells were purified from the spleen of C57BL/6 mice using a mouse B lymphocyte enrichment set (Cat# 557792, BD Biosciences). Briefly, splenocytes in single cell suspension were first incubated with a cocktail of biotinylated antibodies (α-CD4, α-CD43 and α-TER119) that recognize T cells, developing B cells, B-1 cells, activated B cells, antibody secreting plasma cells and erythrocytes, but not resting, mature B cells. After washing, the cells were incubated with the streptavidin particles and the labeled cells were removed by a Cell Separation Magnet. The purity of B cells was >95% as judged by their B220 expression (Supplementary Figure S1A). The majority of the purified B cells were CD23^+^ follicular B (Supplementary Figure S1B) and AA4^−^ mature B cells (Supplementary Figure S1C). Purified primary B cells were cultured in 96 well flat bottom plates (5 × 10^5^/ml, 200μl/well) in triplicate for various time period in medium (RPMI1640 supplemented with 10% FBS, 5 × 10^−5^ M 2-mercaptoethanol, and 100 U of penicillin and streptomycin) alone or in the presence of different concentrations of F(ab′)_2_ α-IgM Abs (Cat# 16-5092, eBioscience). The cells were stained with 7-amino-actinomycin D (7-AAD) and the percentages of viable (7-AAD^low^ FSC^high^) and dead (7-AAD^high^ FSC^low^) cells were analyzed by a FACSVerse flow cytometer (BD Biosciences). Ibrutinib, an inhibitor of the Bruton’s tyrosine kinase (BTK), was purchased from Cosmo Bio (Cat# S2680), Japan, and dissolved in DMSO.

### Analysis of BCR levels

B cells (10^5^) were incubated with PE-conjugated rat α-mouse IgM monoclonal Ab (Cat# 553521, BD Biosciences) on ice and mixed once during the incubation. The cells were then washed twice with 200 µl of FACS buffer (PBS containing 2% FBS and 0.02% sodium azide), suspended in 200 µl of FACS buffer and analyzed with a FACSVerse flow cytometer. The Geo mean fluorescence intensity (MFI) of BCR was used as an index of BCR levels.

### Intracellular staining of phosphorylated SYK (pSYK), ERK (pERK) and AKT (pAKT)

Splenocytes (10^6^) were first incubated on ice for 15 min in 50 µl of FACS buffer (1 x PBS, 2% FBS and 0.02% sodium azide) containing 5 µg/ml of Rat anti-mouse CD16/CD32 (Cat# 553142, BD Biosciences) to block the Fc receptor. The cells were then washed and stained with FITC- or PE-conjugated anti-mouse IgM (Cat# 553408 and Cat# 553521, BD Biosciences). After washing, the cells were fixed and permeabilized with a fixation/permeabilization kit (Cat# 554714, BD Biosciences) according to the manufacturer’s protocol. The cells were then stained with PE-conjugated anti-pSYK (pY348), or Alexa Fluor 647-conjugated anti-pERK1/2 (pT202/pY204) or anti-pAKT (pS473) (BD Biosciences). The levels of pSYK, pERK and pAKT were analyzed in gated IgM^high^ and IgM^low^ population by a FACSVerse flow cytometer as outlined in Supplementary Figure S2.

### Mathematical modeling of the tonic BCR survival signal in B cells

**Figure 1:**
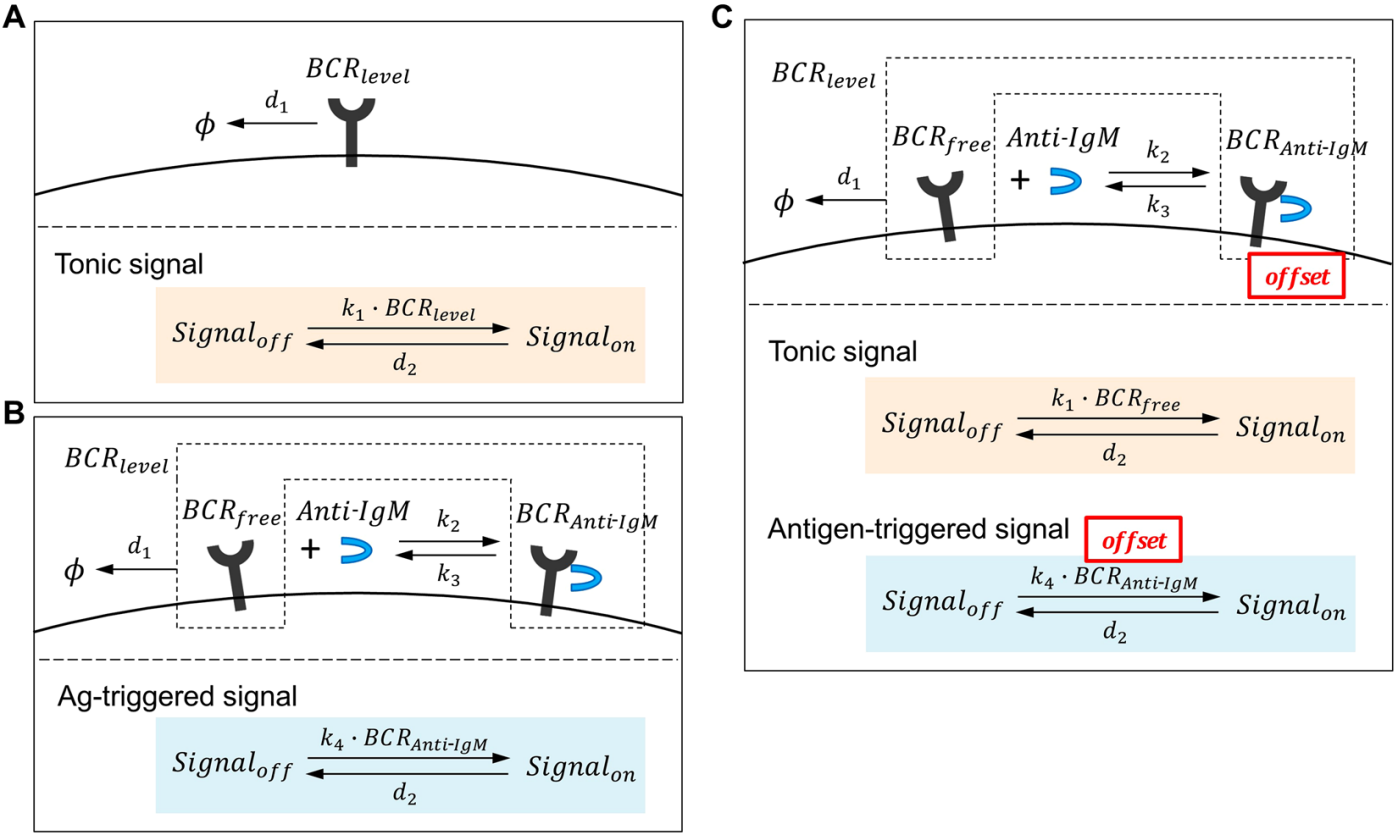
Schematic illustration of BCR tonic signal (**A**), signal induced by crosslinking BCR (**B**) and the combined tonic and activated BCR signals (**C**).

A scheme for BCR level in each cell is illustrated in Fig. 1A. MFI of BCR decreased during culture due to a decrease of BCR level in each B cell. The BCR level in each B cell is different and could be converted to a value between 0 and 1. Therefore, BCR level in individual B cell is shown by the following equation:

1 $$\frac{dBC{R}_{level}}{dt}=-{d}_{1}\cdot BC{R}_{level}$$

where *d*_1_ is the rate of BCR degradation. Our experimental results indicated that the strength of tonic BCR survival signal correlated with BCR levels (MFI). In other words, BCR functioned as if it is an enzyme that catalyzed a chemical reaction to create a tonic survival signal. Accordingly, tonic BCR signal can be expressed by the following reaction:

2 $$Signa{l}_{off}\,\underset{\mathop{\longleftarrow }\limits_{{d}_{2}}}{\overset{{k}_{1}\cdot BC{R}_{level}}{\longrightarrow }}Signa{l}_{on}$$

This chemical reaction means that in the absence of surface BCR expression there is no survival signal generated and the above reaction only proceeds to the left direction (*Signal*_*off*_). The existence of surface BCR constitutively generates a survival signal (*Signal*_*on*_) dependent on BCR levels at a rate constant of *k*_1_. On the other hand, certain amount of the tonic signal is constitutively consumed (signal consumption) at a rate of *d*_2_ to maintain B cell viability. The sum of *Signal*_*on*_ and *Signal*_*off*_ is set to 1. Thus the tonic signal generated from BCR can be expressed as the following equations:

3 $$\begin{array}{c}\frac{dSigna{l}_{on}}{dt}={k}_{1}\cdot Signa{l}_{off}\cdot BC{R}_{level}-{d}_{2}\cdot Signa{l}_{on}\\ Signa{l}_{off}\,\,\,\,=1-Signa{l}_{on}\end{array}$$

In the above equations, if *dSignal*_*on*_ */dt* is smaller than 0, the amount of tonic signal will keep decreasing. We think that when the amount of tonic signal decreased to a certain level (which we call *deadline*), a B cell will die.

### Modeling of Ag-triggered survival signal

A scheme for F(ab′)_2_ α-IgM Abs binding to and release from the surface BCR is illustrated in Fig. 1B and can be expressed as the following equilibrium:

4 $$BC{R}_{free}+Anti \mbox{-} IgM\,\underset{\mathop{\longleftarrow }\limits_{{k}_{3}}}{\overset{{k}_{2}}{\longrightarrow }}BC{R}_{Anti \mbox{-} IgM}$$

where *k*_2_ and *k*_3_ are the rate constant of BCR-anti-IgM binding and disassociation, respectively. *BCR*_*free*_ and *BCR*_*Anti-IgM*_ represent BCR that are not bound or bound by F(ab′)_2_ α-IgM Abs, respectively, and the sum of *BCR*_*free*_ and *BCR*_*Anti-IgM*_ is equivalent to *BCR*_*level*_. When the above reaction reaches an equilibrium, the proportion of the crosslinked BCR (BCR bound by the F(ab′)_2_ α-IgM Abs) among the total BCR can be expressed by the following equation:

5 $$\frac{BC{R}_{Anti \mbox{-} IgM}}{BC{R}_{free}+BC{R}_{Anti \mbox{-} IgM}}=\frac{Anti \mbox{-} IgM}{Anti \mbox{-} IgM+\frac{{k}_{3}}{{k}_{2}}}$$

The unit of F(ab′)_2_ α-IgM Abs is μg/ml. Based on the equation (4), the changes in the crosslinked BCR (*BCR*_*Anti-IgM*_) are therefore expressed by the following equations:

6 $$\begin{array}{ccc}\frac{dBC{R}_{Anti{\textstyle \text{-}}IgM}}{dt} & = & {k}_{2}\cdot Anti{\textstyle \text{-}}IgM\cdot BC{R}_{free}-{k}_{3}\cdot BC{R}_{Anti{\textstyle \text{-}}IgM}\\ BC{R}_{free} & = & BC{R}_{level}-BC{R}_{Anti{\textstyle \text{-}}IgM}\end{array}$$

The above differential equation and the equation (1) lead to the following equation showing the relationship of the concentrations of F(ab′)_2_ α-IgM Abs, time (h) in culture and the proportion of the crosslinked BCR:

7 $$BC{R}_{Anti \mbox{-} IgM}=\frac{{k}_{2}\cdot Anti \mbox{-} IgM(1-{e}^{-({k}_{2}\cdot Anti \mbox{-} IgM+{k}_{3})t})}{{k}_{2}\cdot Anti \mbox{-} IgM+{k}_{3}}$$

The strength of the survival signals (*Signal*_*on*_) correlated with the amount of crosslinked BCR and can be expressed as the following chemical reaction:

8 $$Signa{l}_{off}\,\underset{{d}_{2}}{\overset{{k}_{4}\cdot BC{R}_{Anti{\textstyle \text{-}}IgM}}{\rightleftarrows}}Signa{l}_{on}$$

where *k*_4_ is the rate constant of *Signal*_*on*_ generated by crosslinked BCR and *d*_2_ again is the rate constant of signal consumption required for maintaining the viability of a B cell. Therefore, the survival signal is expressed by the following differential equation:

9 $$\frac{dSigna{l}_{on}}{dt}={k}_{4}\cdot Signa{l}_{off}\cdot BC{R}_{Anti \mbox{-} IgM}-{d}_{2}\cdot Signa{l}_{on}$$

### A Model integrating the tonic and Ag-triggered survival signals

In combining the tonic BCR signal and Ag-triggered signal, we considered two points. First, binding of F(ab′)_2_ α-IgM Abs to BCR induces conformational changes of BCR complex to initiate a cascade of signaling events^1,2^, which we think will simultaneously disrupt the tonic survival signal. Ag-triggered survival signal is thus generated at the expense of tonic signal. Therefore, we assume that the total survival signal (tonic + Ag-triggered − signal consumption) in the presence of BCR crosslinking is expressed by the following differential equation:

10 $$\frac{dSigna{l}_{on}}{dt}={k}_{1}\cdot Signa{l}_{off}\cdot BC{R}_{free}+{k}_{4}\cdot Signa{l}_{off}\cdot BC{R}_{Anti \mbox{-} IgM}-{d}_{2}\cdot Signa{l}_{on}$$

However, the above equation does not take into consideration that there is an activation threshold or offset in Ag-triggered BCR signaling, i.e., if the strength of Ag-triggered signal is below the offset, such a signal will not be converted to an effective survival signal but instead lost^15,16^. We found that crosslinking up to 72% of the total surface BCR did not induce increased B cell survival. More precisely, crosslinking up to 61% of the surface BCR failed to generate a survival signal, and crosslinking 61–72% of the total surface BCR did generate a survival signal but its amount was not greater than the amount of the tonic survival signal lost due to BCR crosslinking, resulting in no increase in B cell survival. Therefore, we assume that the 61% is the true offset for Ag-triggered survival signal and the total survival signal (tonic + [offset Ag-triggered] − signal consumption) can be expressed by the following revised equation:

11 $$\begin{array}{ccc}\frac{dSigna{l}_{on}}{dt} & = & {k}_{1}\cdot Signa{l}_{off}\cdot BC{R}_{free}+{k}_{4}\cdot Signa{l}_{off}\cdot f-{d}_{2}\cdot Signa{l}_{on}\\ f & = & \{\begin{array}{c}BC{R}_{Anti{\textstyle \text{-}}IgM}-BC{R}_{level}\cdot offset\,(if\,BC{R}_{Anti{\textstyle \text{-}}IgM} > BC{R}_{level}\cdot offset)\\ \,0\,(otherwise)\end{array}\end{array}$$

where (*BCR*_*Anti-IgM*_ – *BCR*_*level*_ · *offset*) should be set to 0 if *BCR*_*Anti-IgM*_ is smaller than *BCR*_*level*_ · *offset*. The final scheme showing both the intrinsic tonic and extrinsic Ag-triggered signals is illustrated in Fig. 1C.

### Parameter optimization using weighted least squares method

The *d*_1_, *k*_2_ and *k*_3_ variables were determined using the weighted least squares method to allow the model to best fit with the experimental results. Although the least squares method is normally used for such a modeling, we defined the error value based on the following equation to reduce the influence of the data with large SD and to increase the influence of the data with small SD.

12 $$J=\sum _{i=1}^{n}\frac{{({y}_{i}-f({x}_{i}))}^{2}}{{\sigma }_{i}^{2}}$$

In the above equation, *J* is the error value (sum of the weighted squared difference), *n* is the number of experiments, *y* is the experimental data, *σ* is the SD of the data. The variables of the model (f) were determined to obtain the smallest value of *J*.

### Parameter optimization using genetic algorithm (GA)

GA is a commonly used method for optimizing variables. We used Unimodal Normal Distribution Crossover (UNDX) and Minimal Gap Generation (MGG) methods^21–23^ to calculate the values of parameters *k*_1_, *k*_4_, *d*_2_, *offset* and *deadline*, which cannot be directly determined from the experimental data. To determine *k*_1_, *d*_2_, and *deadline*, we used GA to allow th9 model to best fit with the survival data obtained at 0, 18, 48 and 72 h of culture (a total of 4 data points). First, one hundred individuals with different set of parameters were created as the first generation. Each individual has a randomly created value (0~0.2) for *k*_1_, *d*_2_ and *deadline*, and values of *d*_1_, *k*_2_ and *k*_3_ were determined from experimental data. The *n* + *1* generation was created from *n* generation as outlined in Supplementary Figure S3). The survival rate was calculated for each individual in the *n* + *1* generation and the GA was terminated when the mean value of the sum squared difference of all the individuals was almost the same as that found in an individual with the smallest value of the sum squared difference. The variables of the individual with the smallest value of the sum squared difference was considered to be optimum.

To determine *k*_4_ and *offset*, GA was carried out to allow the modeling to best fit with the actual survival data of 0, 0.3, 3 and 10 µg/mL of F(ab′)_2_ α-IgM Abs at 0, 18, 48 and 72 h (a total of 16 data points). Here again 100 individuals with different parameters were used as the first generation. Each individual was initialized based on the values of *d1*, *k*_2_ and *k*_3_ determined from the experimental data and the values for *k*_1_, *d*_2_, and *deadline* were allowed to change between 50~150% of the previous values, *k*_4_ between 0~0.2 and *offset* between 0~1. GA was then carried out as described in the previous paragraph to search for optimum set of parameters. We generated random numbers using Mersenne Twister method^24^ that can rapidly create high quality pseudorandom numbers. We also carried out GA side by side using 10 different random number seeds and confirmed that very similar optimum parameters were obtained. The parameters calculated from the experimental data and the GA-derived optimum parameters are shown in Table 1.

### Computer simulation of B cell viability

Based on the differential equations obtained for the tonic and Ag-triggered survival signals, we calculated the changes of the following 3 variables in each cell: *BCR*_*level*_, *BCR*_*Anti-IgM*_, *Signal*_*on*_ using the highly accurate fourth-order Runge–Kutta method. The initial value of each variable was defined as in the following: initial *BCR*_*level*_ (MFI) was converted to a value between 0–1 and *BCR*_*Anti-IgM*_ was set to 0. Since the strength of the survival signal before culture correlated with the initial BCR level, *Signal*_*on*_ in each B cell was considered to be the same as the initial *BCR*_*level*_. Each of the 3 variables was calculated for every 0.01 h interval between time 0 to 72 h and a cell was predicted to die when the value of *Signal*_*on*_ dropped below the value of the *deadline*. Simulation was carried out for 200 representative cells at each dose of the F(ab′)_2_-α-IgM Ab and the survival rate was determined based on the predicted fate of these 200 cells. For example, B cell viability was considered to be 40% if 80 out of the 200 cells were alive.

### Data availability

The datasets generated during and/or analyzed during the current study are available from the corresponding author on reasonable request.

## Results

### The strength of tonic BCR signal correlates with BCR levels

**Figure 2:**
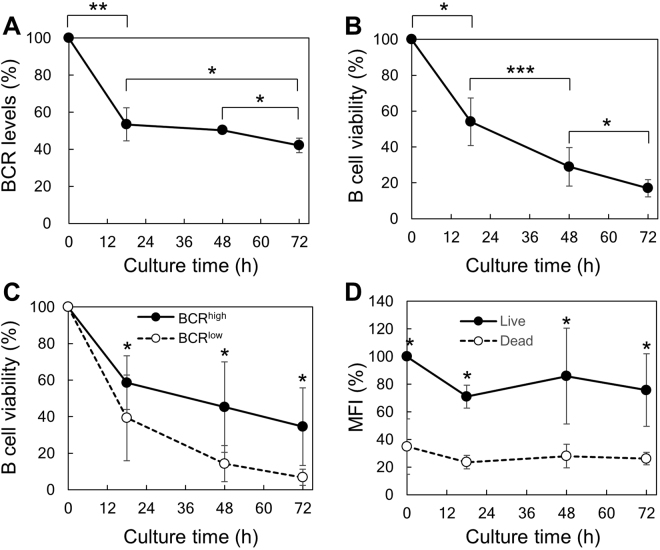
B cell viability correlates with BCR levels. Purified spleen B cells were cultured in the absence of Ag stimulation as described in Materials and Methods and analyzed for their BCR levels and viability at different time points. BCR levels (**A**) and B cell viability (**B**) gradually decreased during culture. (**C**) B cells expressing higher levels of BCR had greatly elevated viability than BCR^low^ cells. B cell viability was analyzed in gated BCR^high^ and BCR^low^ population and was set as 100 at time 0. (**D**) Live B cells had significantly higher BCR levels (shown as % MFI) than did dead B cells. Mean ± SD of 3 independent experiments are shown. *p < 0.05; **p < 0.01; ***p < 0.005 (paired *t* test).

We purified spleen B cells and first analyzed the changes of their BCR levels during *in vitro* culture in the absence of extrinsic stimuli. As shown in Fig. 2A, BCR levels gradually decreased during culture. BCR levels are normally maintained by a dynamic equilibrium of at least the following 4 processes: the assembly and transport of newly synthesized BCR, internalization of the surface BCR, recycling of the internalized BCR back to the cell surface, and degradation of the internalized BCR^25^. In the absence of extrinsic stimuli that induce active gene transcription and protein translation, we think that the amount of the newly synthesized BCR is smaller than the amount degraded, leading to a gradual decrease of surface BCR levels. B cell viability concomitantly decreased during the culture, with some cells died at early time point whereas some cells survived even after 3 days (Fig. 2B). Interestingly, B cells expressing high levels of BCR (BCR^high^) had significantly elevated viability than BCR^low^ cells (Fig. 2C). Conversely, MFI of BCR in live cells was consistently >2-fold that in dead cells (Fig. 2D). These results suggested a positive correlation between BCR levels and B cell viability. Notably, BCR levels decreased only moderately from 18 h to 72 h (Fig. 2A), in contrast to a greater decrease in B cell viability during the same period (Fig. 2B). We think that there is a time lag between the reduction in BCR levels and the decrease in B cell viability.

**Figure 3:**
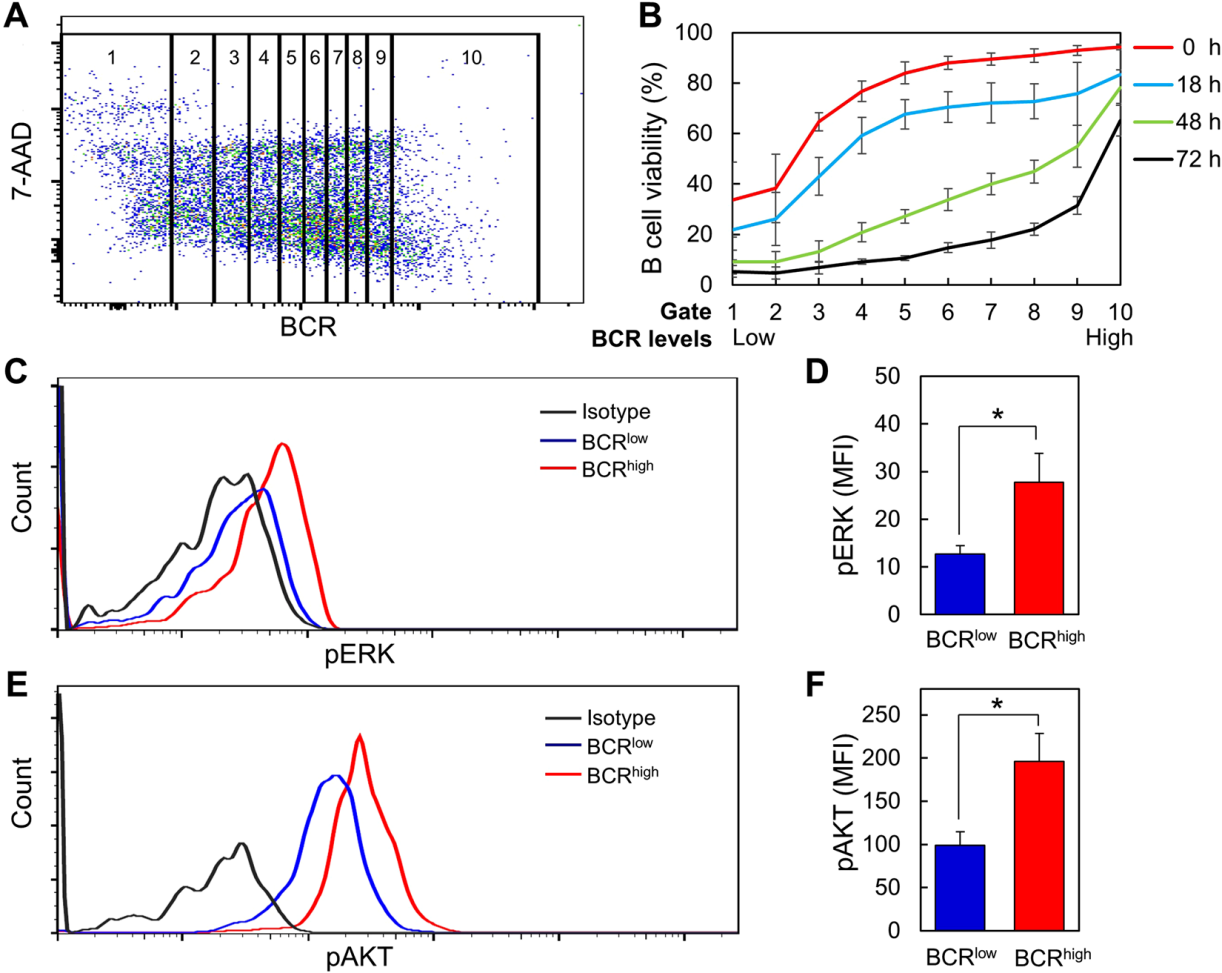
B cells expressing higher levels of BCR have elevated viability and tonic signal than B cells expressing low levels of BCR. (**A**) B cells were gated into 10 population based on their BCR levels. Each population contained the same numbers of B cells. (**B**) Viability of each gated B cell population before culture (0 h) and at 18 h, 48 h and 72 h after culture in medium alone. Levels of phosphorylated ERK (pERK) (**C** and **D**) and pAKT (**E** and **F**) in gated BCR^low^ and BCR^high^ population are shown. Representative FACS profiles (**C** and **E**) and mean ± SD of 3 independent experiments (**D** and **F**) are shown. Gating strategies are outlined in Supplementary Figure S2. *p < 0.05 (paired *t* test).

To confirm the results shown in Fig. 2, we further gated B cells into 10 subpopulations based on their BCR levels (Fig. 3A) and analyzed their survival during culture. The results demonstrated that B cell viability positively correlated with BCR levels at each time point during culture (Fig. 3B). Moreover, we found that BCR^high^ cells had elevated levels of the phosphorylated ERK (pERK) (Fig. 3C and D) and AKT (pAKT) (Fig. 3E and F) than BCR^low^ cells. In contrast, SYK phosphorylation was undetectable both in BCR^high^ and BCR^low^ cells (Supplementary Figure S2). Notably, BCR^high^ cells had a remarkable increase in the levels of pAKT over the background (isotype) but only showed a moderate increase in the levels of pERK. AKT Phosphorylation has been shown to mediate B cell survival by promoting FOXO1 degradation^3,19^. These results collectively indicate that BCR^high^ cells have stronger tonic survival signals than do BCR^low^ cells.

### Tonic BCR signal contributes to the survival of B cells cultured in the absence of Ag stimulation

**Figure 4:**
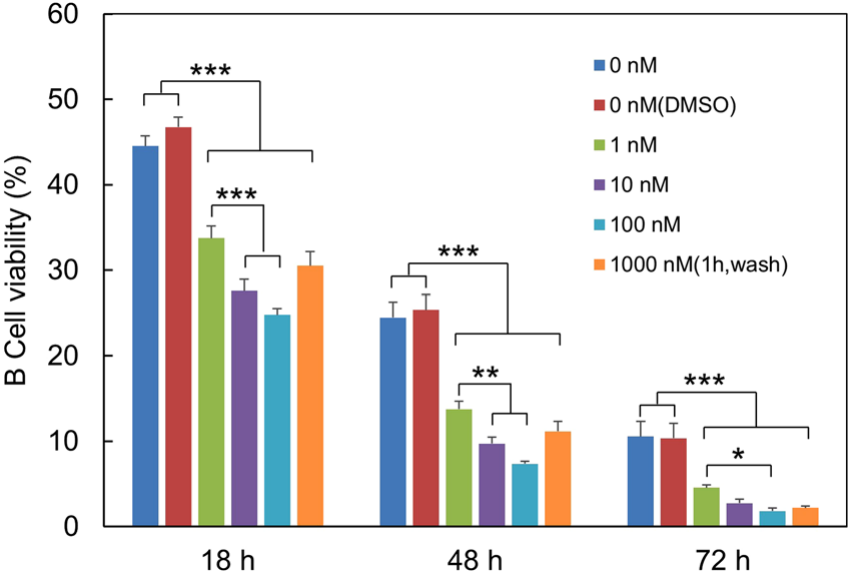
Inhibition of BCR signaling results in decreased B cell survival. Purified spleen B cells were cultured in medium in the absence or presence of 1, 10 or 100 nM of Ibrutinib, an inhibitor of BTK. Alternatively, B cells were incubated with 1 µM of Ibrutinib for 1 h, washed twice to remove Ibrutinib, and then cultured in medium for various time periods. The cells were analyzed for their viability at different time points. Mean ± SEM of 3 independent experiments are shown. *p < 0.05; **p < 0.01; ***p < 0.005 (One-way Anova).

To verify that the survival of primary B cells cultured in the absence of Ag stimulation was dependent on tonic BCR signal, we compared the kinetics of B cell survival in the presence or absence of Ibrutinib, an inhibitor of BTK that plays a critical role in BCR signaling. As shown in Fig. 4, in the continuous presence of Ibrutinib (1 nM-100 nM), but not DMSO used to dissolve Ibrutinib, B cell viability decreased significantly during culture. Incubation of B cells with a higher dose of Ibrutinib (1000 nM) for only 1 h, followed by wash of B cells with the culture medium, also resulted in a significant decrease in B cell viability (Fig. 4). Furthermore, pAKT levels were significantly decreased in the presence of 100 nM of Ibrutinib (Supplementary Figure S4). These results indicate that the survival of B cells cultured in the absence of Ag stimulation is dependent on tonic BCR signal and inhibition of this tonic signal by a BTK inhibitor results in decreased survival.

### Low doses of F(ab′)_2_ α-IgM Abs do not enhance but rather reduce B cell survival

**Figure 5:**
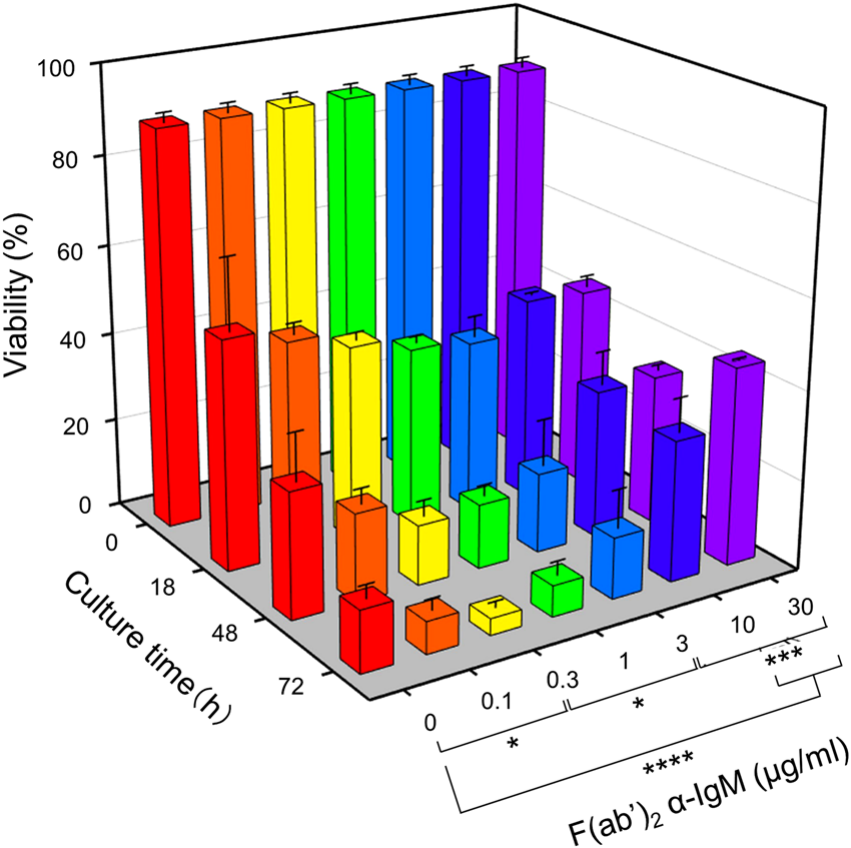
A 3D illustration showing that stimulation with low doses of F(ab′)_2_ α-IgM Abs resulted in decreased B cell viability compared with unstimulated cells. Purified spleen B cells were cultured in the absence or presence of various concentrations of F(ab′)_2_ α-IgM Abs and analyzed for their viability at different time points. Mean ± SD of 2 independent experiments are shown. *p < 0.05; ***p < 0.005; ****p < 0.001 (One-way Anova).

Crosslinking BCR on mature B cells with F(ab′)_2_ α-IgM Abs, which mimic Ag stimulation, initiates signal cascades that eventually lead to B cell survival and activation. It is unclear, however, how the Ag-induced survival signal and the tonic signal are integrated to allow appropriate B cell responses. We first analyzed B cell viability in the presence of different doses of F(ab′)_2_ α-IgM Abs. As shown in Fig. 5, F(ab′)_2_ α-IgM Abs at 10 and 30 μg/ml enhanced B cell viability as compared with B cells cultured in medium alone (0 μg/ml) at both 48 h or 72 h. These observations are consistent with the notion that crosslinking BCR on mature B cells triggers a survival and activation signal. Intriguingly, low doses of F(ab′)_2_ α-IgM Abs (0.1 to 3 μg/ml) did not enhance, but rather decreased, B cell viability, when compared with non-stimulated B cells (Fig. 5).

### B cell survival is enhanced only when most of their BCR are occupied by the F(ab′)2 α-IgM Abs

**Figure 6:**
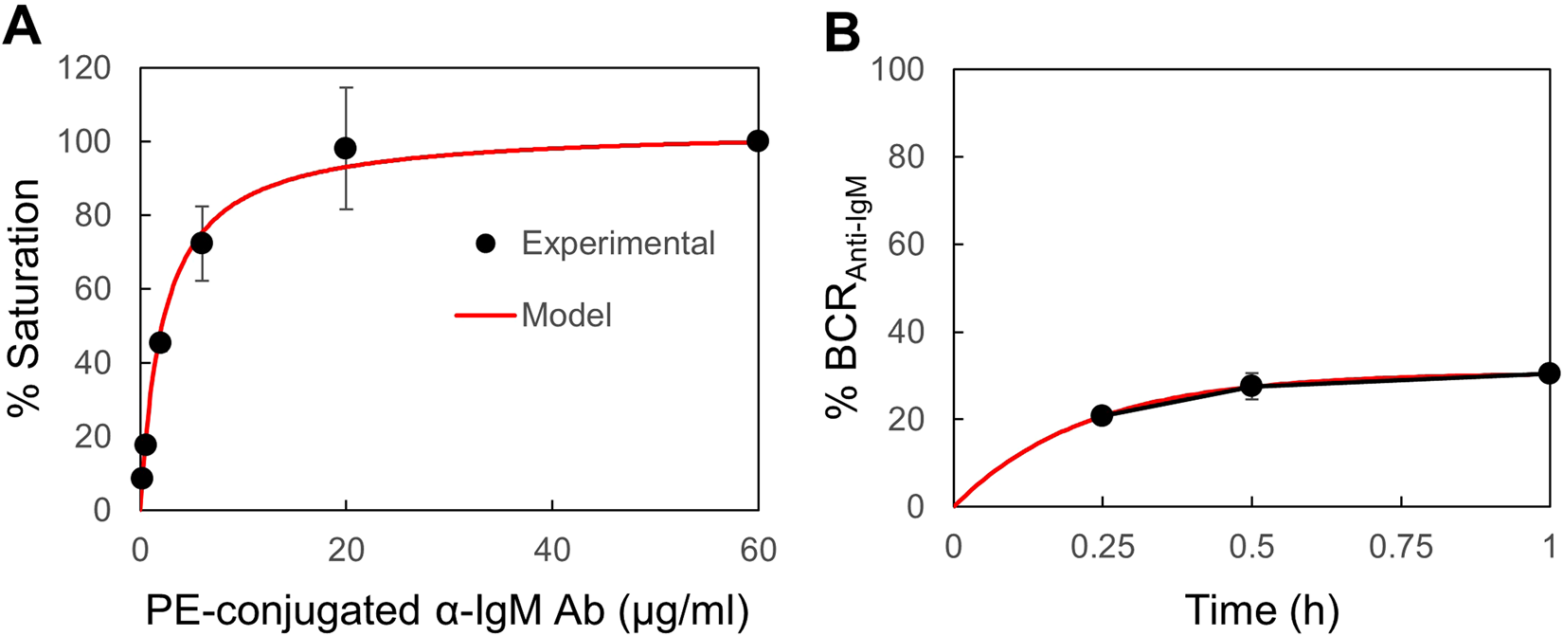
Determination of the α-IgM Ab concentrations that saturate the surface BCR. (**A**) Purified spleen B cells were stained with different concentrations of a PE-conjugated α-IgM Ab and analyzed for MFI by FACS. MFI obtained with 60 µg/ml of PE-conjugated α-IgM Ab was set to 100. (**B**) Proportion of crosslinked BCR in the presence of 1 μg/ml of PE-conjugated α-IgM Ab. Solid circle, actual experimental data; Red line, simulation results. Mean ± SD of 2 independent experiments are shown.

We next investigated the mechanism underlying the nonlinear response in B cell survival induced by F(ab′)_2_ α-IgM Abs, as shown in Fig. 5. We hypothesized that the overall strength of signals induced by F(ab′)_2_ α-IgM Abs should correlate with the amount of cell surface BCR that are crosslinked by these Abs. We therefore stained B cells with different concentrations of PE-conjugated α-IgM Ab to estimate the proportion of the surface BCR that are bound by F(ab′)_2_ α-IgM Abs. We stained 10^5^ cells in 200 μl of medium in a 96 well flat bottom plate, which was the same conditions as we used for cell culture, to correctly estimate the proportion of crosslinked BCR at different concentrations of F(ab′)_2_ α-IgM Abs. Since the molecular weight of the PE-conjugated α-IgM Ab (180–200-kDa) is roughly twice that of the F(ab′)_2_ α-IgM Abs (100-kDa) which lack the Fc portion of the IgG, we stained B cells with 0.2, 0.6, 2, 6, 20 and 60 μg/ml of PE-conjugated α-IgM Ab, which had roughly the same molar concentration as 0.1, 0.3, 1, 3, 10 and 30 μg/ml of the F(ab′)_2_ α-IgM Abs, respectively. As shown in Fig. 6A, the MFI almost reached a plateau at 20 and 60 μg/ml of PE-labeled α-IgM Ab (solid circles), which were defined as 100% of saturation. At 6 μg/ml or lower concentrations of PE-labeled α-IgM Ab, only up to 72% of the cell surface BCR were occupied (solid circles). These observations suggest that B cell survival can be enhanced only when most of their BCR were crosslinked by the F(ab′)_2_ α-IgM Abs.

### Mathematical modeling of the tonic BCR survival signal in B cells

**Figure 7:**
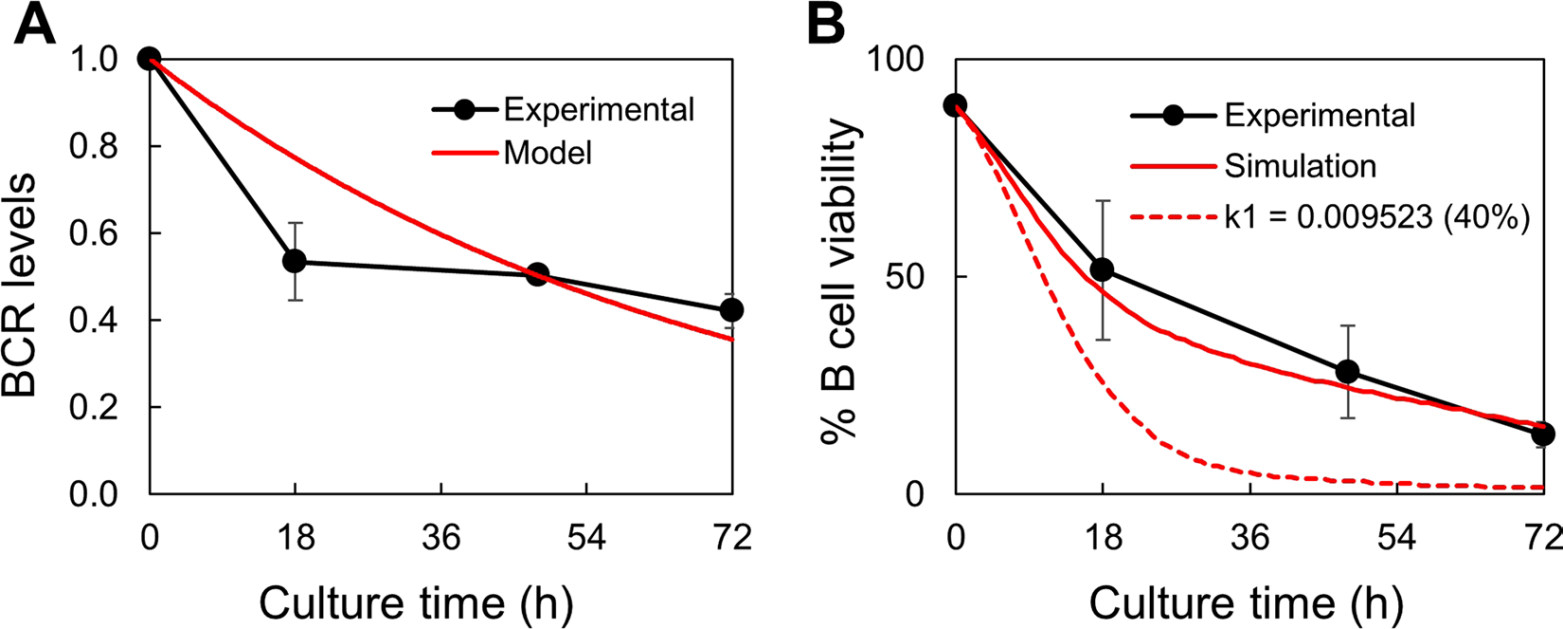
Simulation of BCR levels and B cell viability. (**A**) Decrease in BCR levels during culture. The experimental data (same as Fig. 2A) and simulation are shown by black and red lines, respectively. (**B**) B cell viability in the absence of external stimuli. Actual data (same as Fig. 5, 0 µg/ml of F(ab′)_2_ α-IgM Abs) and simulation results are indicated by black and red lines, respectively. Dotted red line, simulation results when *k*_1_ was set to ~40% of the original value.

To better understand how the tonic signal and Ag-triggered signal are integrated to regulate B cell survival and activation, we first performed mathematical modeling of the tonic BCR signal in B cells cultured in the absence of F(ab′)_2_ α-IgM Abs. Our experimental results indicated that the strength of tonic signal positively correlated with BCR levels and therefore we simply used MFI of BCR as the generator of tonic signal. We assumed that the decreased MFI in cultured B cells was due to a decrease in the BCR level on each B cell and that the tonic signal in each B cell correlated with its BCR level. The distribution of BCR levels of 8342 cells before culture (0 h) (Supplementary Figure S5) indicated that BCR levels in primary B cells differed by more than 1000-fold. To determine *d*_1_ in equation 1, BCR level at 0 h was set to 1 and based on the BCR levels at 18 h, 48 h and 72 h (Fig. 2A), *d*_1_ was determined to be 0.01435 [/h] using the weighted least squares method. The simulation of the decrease in BCR levels is shown in Fig. 7A (red line), which indicated that BCR levels decreased from 1 at 0 h to 0.3558 at 72 h, equivalent to a 64% reduction.

We next used GA to calculate the values of *k*_1_, *d*_2_ and *deadline* in the absence of stimuli (equation 2) to allow the model to best fit with the actual survival data shown in Fig. 5 (0 µg/ml of F(ab′)_2_ α-IgM Abs). To facilitate the simulation of B cell survival, we selected 200 cells with their distribution of BCR levels similar to that of the parental 8342 cells. The highest fluorescence intensity of BCR (67558) was converted to 1, and the average and the median of BCR levels became 0.07460 and 0.04360, respectively. Optimum parameters obtained with 10 different seeds were averaged and *k*_1_, *d*_2_ and *deadline* were determined to be 0.02381 [/h], 0.1239 [/h] and 0.01034, respectively. The survival curve depicted from these parameters is shown in Fig. 7B (solid red line), which well matched the actual data (Fig. 7B, black line). If we set the value of *k*_1_ (the parameter for tonic signal) to 0.009523 [/h] (equivalent to ~40% of the original value of *k*_1_), the survival curve (Fig. 7B, dotted red line) appeared very similar to that obtained in the presence of 100 nM of the BTK inhibitor Ibrutinib (Fig. 4, light blue column), suggesting that the generation of tonic signal decreased by 60% in the presence of 100 nM of Ibrutinib.

### Modeling of the survival signal generated by stimulation with F(ab′)_2_ α-IgM Abs

Based on the results shown in Fig. 6A (solid circles) and using weighted least squares method, the ratio of *k*_3_/*k*_2_ was determined to be 1.132. The result of simulation is shown in Fig. 6A (red line) where the proportion of the crosslinked BCR at 60 μg/ml of PE-conjugated Ab was set as 100%. To determine the values of *k*_3_ and *k*_2_, we analyzed the changes in the proportion of crosslinked BCR. An example is shown in Fig. 6B where the proportion of the crosslinked BCR reached a plateau in 1 h in the presence of 1 μg/ml of PE-conjugated α-IgM Ab. Based on the experimental data shown in Fig. 6B (solid circles), *k*_2_ and *k*_3_ was determined to be 2.779 [ml/μg/h] and 3.145 [/h], respectively, using the weighted least squares method. The values of *k*_2_ and *k*_3_ indicated that 95% of the BCR were saturated in 30 min in the presence of 1 μg/ml of the F(ab′)_2_ α-IgM Abs. Theoretically, only 82% of the BCR would be crosslinked in 30 min in the presence of 0.1 μg/ml of the F(ab′)_2_ α-IgM Abs whereas 99% of the BCR would be crosslinked in just 9 min in the presence of 10 μg/ml of the F(ab′)_2_ α-IgM Abs.

### Integrating the tonic BCR signal and the survival signal generated by stimulation with F(ab′)_2_ α-IgM Abs

**Figure 8:**
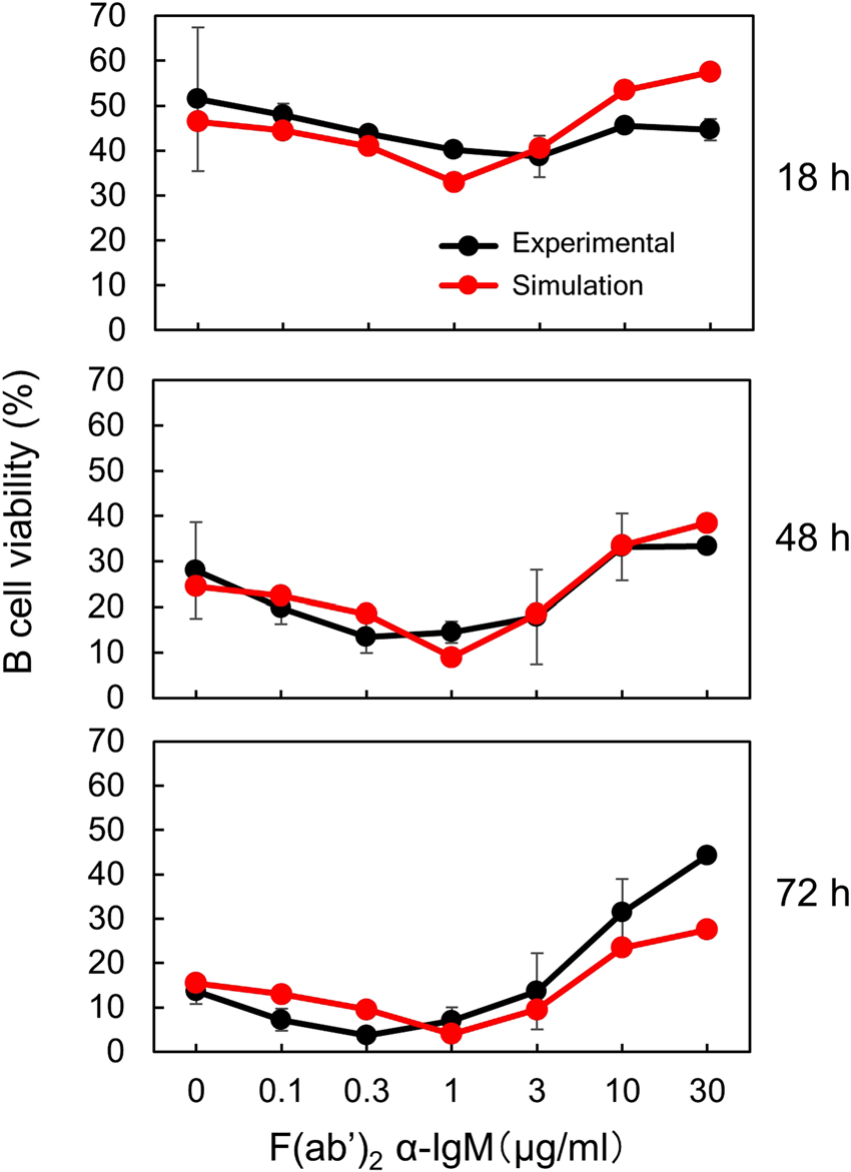
A mathematical model integrating the tonic and Ag-triggered survival signals correctly predicts the viability of B cells cultured in the presence of different doses of F(ab′)_2_ α-IgM Abs for different time periods. Actual experimental data (adopted from Fig. 5, black) and simulation results (red) are shown.

Crosslinking BCR with F(ab′)_2_ α-IgM Abs is known to trigger signal cascades that eventually lead to B cell survival and activation. The strength of the signal is determined by the concentration of F(ab′)_2_ α-IgM Abs. At 3 μg/ml or lower doses of F(ab′)_2_ α-IgM Abs (equivalent to <6 μg/ml of the PE-conjugated α-IgM Ab), only up to 72% of the BCR were crosslinked (Fig. 6A, solid circles) and in such cases a survival signal cannot be generated (Fig. 5). More precisely, crosslinking up to 61% of the surface BCR failed to generate a survival signal, and crosslinking 61–72% of the total surface BCR did generate a survival signal but its strength was not greater than the tonic survival signal lost due to BCR crosslinking, resulting in no increase in B cell survival. At 10 and 30 μg/ml of F(ab′)_2_ α-IgM Abs (equivalent to 20 and 60 μg/ml of PE-conjugated α-IgM Ab), almost all the BCR were crosslinked (Fig. 6A, solid circles), resulting in the generation of survival signals (Fig. 5). Next, we used GA to determine *k*_4_ and *offset* in the presence of F(ab′)_2_ α-IgM stimulation based on equations 1, 6 and 11. We used the data of 0, 0.3, 3 and 10 µg/ml shown in Fig. 5 and obtained following results: *k*_1_, 0.02980; *d*_2_, 0.1543; *deadline*, 0.01032; *k*_4_, 0.1294; *offset*, 0.6097 (Summarized in Table 1). Based on these parameters, we calculated viability of B cells cultured in the presence of different concentrations of F(ab′)_2_ α-IgM Abs for different times (Fig. 8, red line), which correlated well with the actual experimental data (Fig. 8, black line). These results suggest that our model successfully integrated the tonic and Ag-triggered signals and correctly predicted the survival probability of primary B cells. In particular, the reduced B cell survival at low doses of F(ab′)_2_ α-IgM Abs was very well recapitulated in the simulation.

**Table 1:** Parameters used in modeling the tonic and Ag-triggered survival signals.

Parameter	Initial value range	Optimum value ± SD (%)^a^	Unit
*d*_1_	—	0.01435	/h
*k*_1_	0–0.2	0.02980 ± 0.4695%	/h
*k*_2_	—	2.779	ml/μg/h
*k*_3_	—	3.145	/h
*k*_4_	0–0.2	0.1294 ± 1.795%	/h
*d*_2_	0–0.2	0.1543 ± 0.3359%	/h
*offset*	0–1.0	0.6097 ± 0.8074%	—
*deadline*	0–0.2	0.01032 ± 0.7700%	—

^a^Parameters without initial value range were calculated based on experimental data and those with initial value range were optimized using genetic algorithm. The variations in the optimal value in the 10 seeds of random numbers are shown.

## Discussion

Although BCR constitutively transmits a tonic survival signal in the absence of Ag stimulation, it is difficult to analyze its nature using conventional biochemical approaches. To overcome this difficulty, we have combined actual experiments with mathematical modeling to investigate its strength, regulation and relationship with the Ag-triggered survival signal. In the present study, we have successfully built a mathematical model that integrates tonic BCR signal and signal induced by BCR crosslinking. Our model reveals that the rate constant for the generation of tonic signal (*k*_1_) and Ag-triggered signal (*k*_4_) is 0.02980 and 0.1294, respectively. In other words, crosslinking a BCR generates a signal that is 4.3 (0.1294/0.02980) times as strong as the tonic signal generated from a free BCR. To our knowledge, this is the first attempt to determine the strength of tonic BCR signal relative to the signal generated from crosslinked BCR. This model also allows us to predict the survival probability of a B cell based on its initial BCR level and the strength and duration of BCR crosslinking and fits very well with the mechanisms that regulate B cell tolerance.

Another intriguing finding in the present study is that a survival signal is not generated even when 61% of the surface BCR is crosslinked by F(ab′)_2_ α-IgM Abs. We think that the strength of the signal generated by crosslinking 61% of the surface BCR corresponds to the threshold (or offset) for Ag-triggered signaling. The existence of such a threshold is important for B cells not to respond to stimulation by self Ag. B cell development proceeds in an ordered fashion in the bone marrow (BM). Committed B cell progenitors rearrange their Ig heavy and then light chain genes to become IgM^+^ immature B cells. Immature B cells that react strongly with self Ag are deleted in BM, a mechanism termed central B cell tolerance^1^. Those that do not react with self Ag strongly are allowed to exit BM and migrate to the secondary lymphoid tissues such as spleen and lymph node where they become mature B cells. Therefore, mature B cells in general do not react strongly with self Ag. The existence of a threshold in BCR signaling can effectively prevent mature B cells from being activated by self Ag and allow a robust response of mature B cells against foreign Ag that can crosslink BCR with high affinity and/or high avidity.

It should be noted that this threshold applies only when BCR alone is stimulated. If a B cell simultaneously receives stimulation through other receptors such as CD40 or cytokine receptors, crosslinking most of the surface BCR is no longer a prerequisite for inducing a survival signal. Such a situation occurs when B cells are stimulated by foreign Ag. In this case, foreign Ag-specific T cells will provide help for B cells by upregulation of CD40 ligand that can bind to CD40 on B cells and by secretion of cytokines such as IL-4. Therefore, the existence of such a threshold does not inhibit B cell activation against foreign Ag but only prevents B cell response against self Ag in which case B cells cannot receive T cell help as self Ag-specific T cells are normally deleted in the thymus during T cell development. In the present study, we focused on the survival signal generated by BCR alone and in this case the existence of a high threshold for inducing B cell survival functions to prevent B cell activation by self Ag stimulation.

We found that BCR^high^ cells had significantly elevated levels of pAKT compared with BCR^low^ cells. In contrast, both BCR^high^ and BCR^low^ cells had similarly undetectable levels of pSYK. Using genetic approaches, Srinivasan *et al*. have elegantly demonstrated that tonic BCR survival signal is predominantly mediated by PI3 kinase, which targets AKT for phosphorylation^22^. AKT phosphorylation promotes B cell survival by inactivating transcription factor FOXO1^3^. The elevated AKT phosphorylation in BCR^high^ cells is therefore consistent with the increased survival of these cells. It has also been demonstrated that canonical NF-κB signaling, or MEK1 and Rac1 activation, fails to rescue the survival of B cells lacking BCR^22^. MEK is known to phosphorylate ERK^3^ and we found that BCR^low^ cells had barely detectable levels of pERK and BCR^high^ cells only had a moderate increase of pERK over the background (isotype). The limited amount of pERK in BCR^high^ cells is also in agreement with the previous finding that MEK-ERK MAP kinase pathway does not mediate B cell survival^22^. We think that the levels of surface BCR and intracellular pAKT are two important parameters reflecting the strength of tonic BCR signal.

We have used primary mature B cells purified from mouse spleen. More than 90% of the purified spleen B cells are follicular B cells and about 5–10% are marginal zone B cells (MZB). These MZB express higher levels of BCR than do follicular B cells and are thought to have a tendency to react with self Ag and differentiate into Ab secreting plasma cells^26^. It would therefore be interesting to analyze the survival of MZB in the absence or presence of different doses of F(ab′)_2_ α-IgM Abs and build a mathematical model predicting their fate. Another important B cell subpopulation are B-1 cells, which are localized in the peritoneal and plural cavities and secrete natural Abs even in the absence of BCR crosslinking^27^. It remains to be investigated how the tonic and Ag-triggered survival signals are regulated in B-1 cells. Development of mathematical models predicting the survival of these different B cell subsets should provide important insights in understanding their differential responses to foreign and self Ag stimulation. Moreover, memory B cells, which can be rapidly activated by Ag stimulation, normally express isotype-switched IgG-BCR. The IgG-BCR has unique structural and biological properties^28^ and it would greatly help understand memory B cell responses by establishing a mathematical model integrating tonic and Ag-triggered signals from IgG-BCR.

The tonic BCR signal is important for the survival of not only normal B cells but also B lymphoma cells. Although there are many subtypes of B lymphomas, an intriguing common feature is that all types of B lymphomas require BCR signals to gain advantages in their tumorigenesis *in vivo* ^10,11^ and the BTK inhibitor has demonstrated clinical benefit for several types of B cell lymphomas. It remains to be investigated how the relatively “weak” tonic BCR signal is critical for B cell malignancies. One possibility is that although B lymphoma cells have acquired oncogenic mutations that allow their uncontrolled proliferation, they still rely on BCR signals for their survival. The results of the current study suggest that inhibition of BCR expression might lead to decreased tonic BCR signal in B lymphomas. Mathematical modeling of the tonic BCR signal in normal and malignant human B cells should provide important clues for understanding the pathogenesis of B lymphomas and for developing effective therapies.

## Electronic supplementary material

Supplementary data
