# Supplementary material for: A model integrating tonic and antigen-triggered BCR signals to predict the survival of primary B cells
Source: Sci Rep. 2017 Nov 2;7:14888. doi: 10.1038/s41598-017-13993-x (PMC5668375; doi:10.1038/s41598-017-13993-x)
Supplement: Supplementary file 1 — Supplementary data [file 41598_2017_13993_MOESM1_ESM.pdf]

Supplementary Figure S1

A model integrating tonic and antigen-triggered BCR signals to predict the survival of primary B cells

Shoya Yasuda, Yang Zhou, Yanqing Wang, Masayuki Yamamura, and Ji-Yang Wang

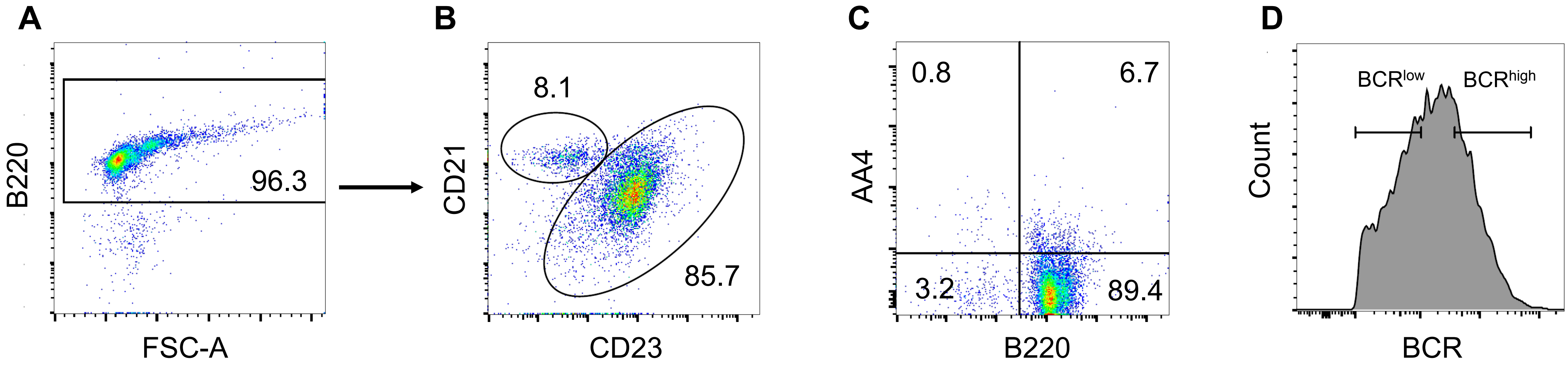

Supplementary Figure S1. Mature B cells were purified from mouse spleen as described in Materials and Methods and analyzed for their expression of B220 (**A**), CD23 and CD21 in gated B220^{+} cells (**B**), B220 and AA4 (**C**), and BCR (**D**). The gates for BCR^{low} and BCR^{high} population are shown in **D**.

Supplementary Figure S2

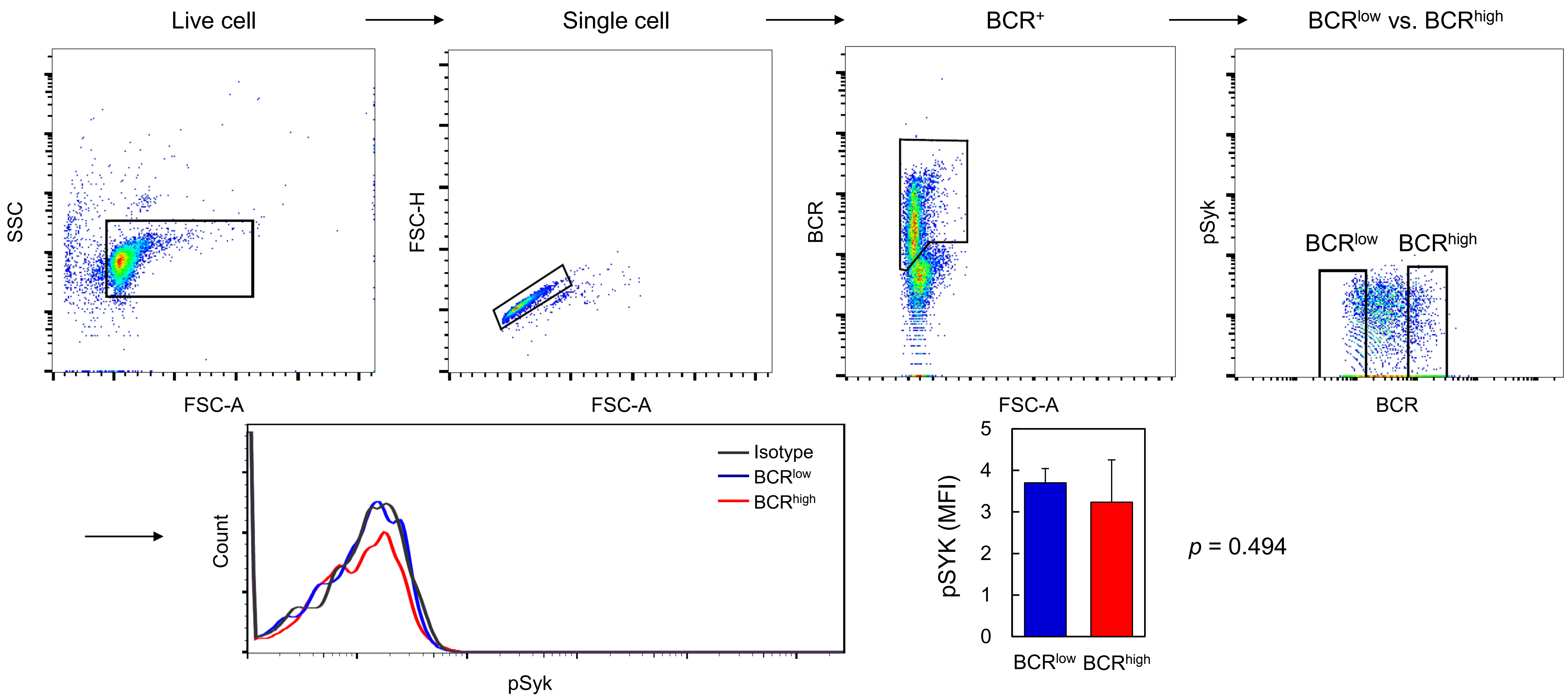

Supplementary Figure S2. Gating strategies for the analysis of phosphorylated SYK (pSYK).

Supplementary Figure S3

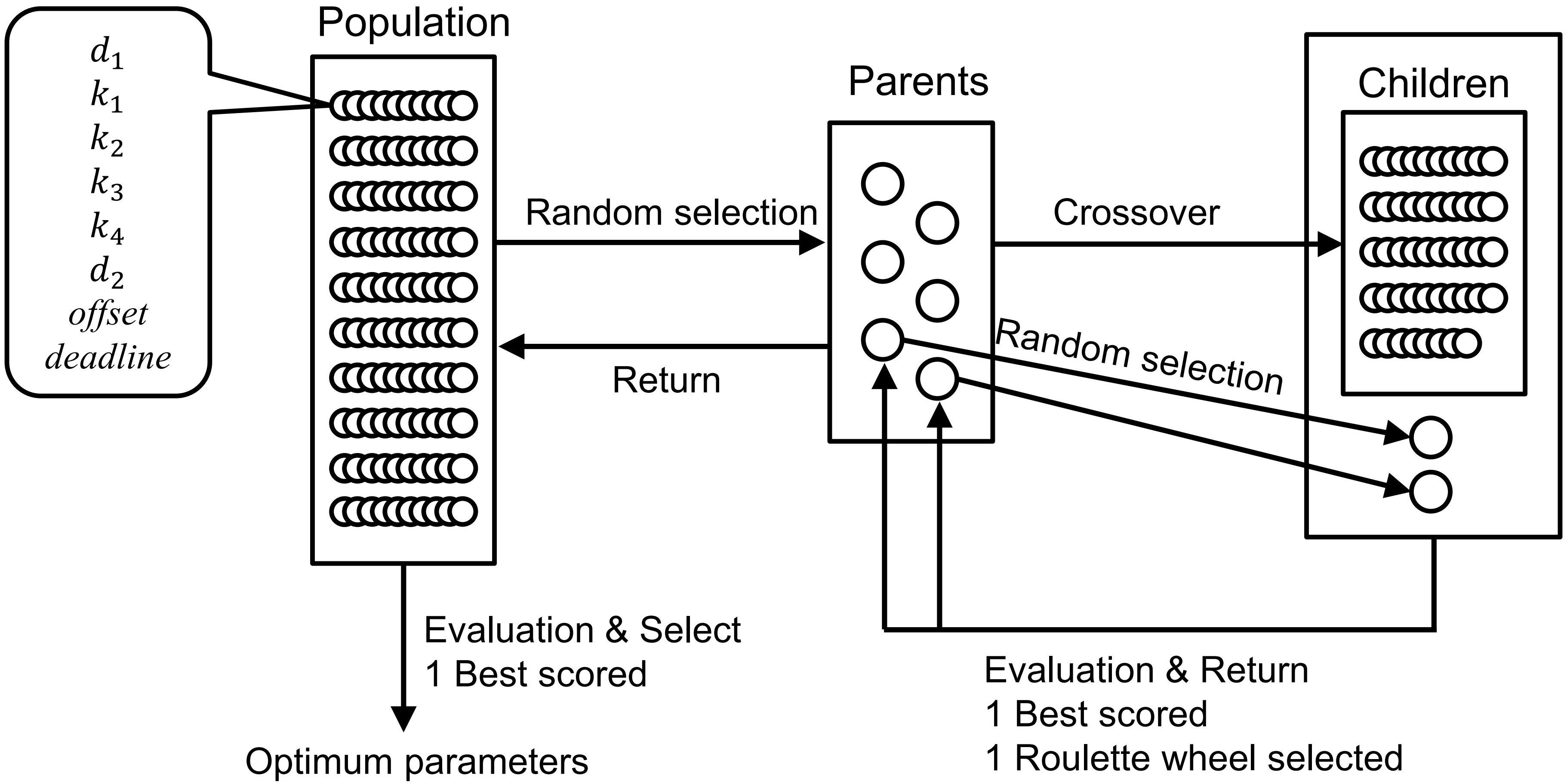

Supplementary Figure S3. A schematic model of the genetic algorithm (GA). Six individuals randomly selected from the original population were used to generate a family containing 48 children by crossover, which was evaluated and selected together with the 2 individuals of the parents. Each individual has a different set of parameters and is shown as an open circle.

Supplementary Figure S4

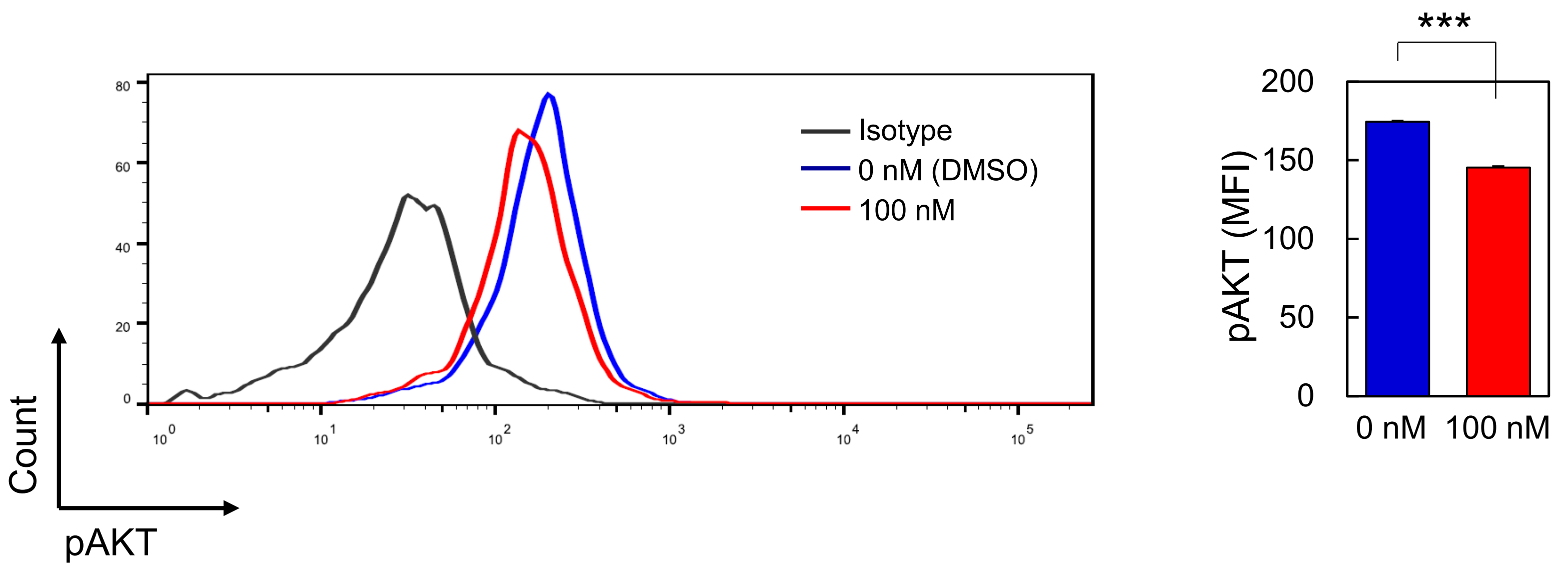

Supplementary Figure S4. pAKT levels were significantly reduced in the presence of 100 nM of Ibrutinib compared with DMSO. Left panel, representative FACS profiles; Right panel, Mean ± S.D. of 2 experiments. *** $p < 0.005$.

Supplementary Figure S5

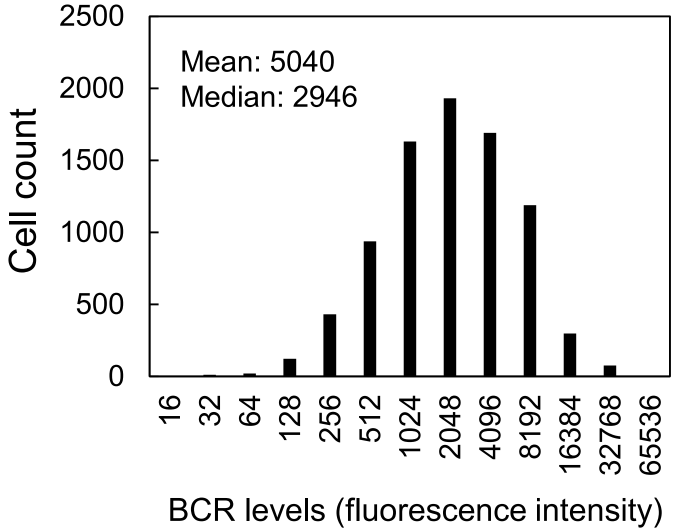

Supplementary Figure S5. Distribution of BCR levels of 8342 cells.
